# Blockade of GCH1/BH4 Axis Activates Ferritinophagy to Mitigate the Resistance of Colorectal Cancer to Erastin-Induced Ferroptosis

**DOI:** 10.3389/fcell.2022.810327

**Published:** 2022-02-10

**Authors:** Qian Hu, Wanhui Wei, Daiqian Wu, Fengxing Huang, Mengting Li, Wenjie Li, Jingwen Yin, Yanan Peng, Yuanyuan Lu, Qiu Zhao, Lan Liu

**Affiliations:** ^1^ Department of Gastroenterology, Zhongnan Hospital of Wuhan University, Wuhan, China; ^2^ Hubei Clinical Center and Key Lab of Intestinal and Colorectal Diseases, Wuhan, China; ^3^ Department of Cardiology, Zhongnan Hospital of Wuhan University, Wuhan, China

**Keywords:** colorectal cancer, ferroptosis, GCH1, tetrahydrobiopterin, ferritinophagy, erastin

## Abstract

Ferroptosis, a type of cell death triggered by excessive accumulation of iron-dependent lipid peroxidation, possesses an excellent potential in cancer treatment. However, many colorectal cancer (CRC) cell lines are resistant to ferroptosis induced by erastin and RSL3, the classical ferroptotic inducers. Moreover, the underlying mechanism of resistance remains poorly elucidated. This study sought to discover the major factor contributing to ferroptosis resistance in CRC. The study findings will help design strategies for triggering ferroptosis for application in individualized tumor therapy. Here, we show that tetrahydrobiopterin (BH4) determines the sensitivity of CRC cells to ferroptosis induced by erastin. GTP cyclohydrolase-1 (GCH1) is the first rate-limiting enzyme of BH4. Genetic or pharmacological inhibition of GCH1 decreased BH4 and assisted erastin in cell death induction, lipid peroxidation enhancement, and ferrous iron accumulation. BH4 supplementation completely inhibited ferroptotic features resulting from GCH1 knockdown. Unexpectedly, GCH1 knockdown failed to enhance RSL3-induced cell death in CRC. Mechanistically, GCH1 knockdown drastically activated ferritinophagy during erastin treatment rather than RSL3 treatment. Administration of an autophagy inhibitor reversed erastin resistance in GCH1-knockdown cells. GCH1 inhibitor and erastin co-treatment *in vivo* synergistically inhibited tumor growth in CRC. Overall, our results identified GCH1/BH4 metabolism as a burgeoning ferroptosis defense mechanism in CRC. Inhibiting GCH1/BH4 metabolism promoted erastin-induced ferroptosis by activating ferritinophagy, suggesting that combining GCH1 inhibitors with erastin in the treatment of CRC is a novel therapeutic strategy.

## Introduction

Colorectal cancer (CRC) is the second deadliest cancer worldwide (Bray et al., 2018). The global burden of CRC is expected to rise by 60% to more than 2.2 million new cases and 1.1 million deaths by 2030 (Arnold et al., 2017). Although immunotherapy and molecular-targeted therapy are developing, chemotherapy is still a critical and conventional treatment for CRC (Benson et al., 2018; Parseghian et al., 2019; Xie et al., 2020). However, chemotherapy is often limited by cancer cell resistance to chemotherapy drugs. Apoptosis suppression is a common drug-resistance mechanism that frequently occurs during or before treatment (Jeught et al., 2018; Wang, 2020). Therefore, an urgent need exists to explore novel cell death mechanisms and their potential application in CRC treatment.

Ferroptosis is a novel non-apoptotic form of regulated cell death, characterized by iron accumulation and lipid peroxidation (Tang et al., 2021). Since iron overload exists in various cancer cells, it makes ferroptosis induction a promising strategy in cancer therapy (Torti et al., 2018). Several studies have revealed that erastin and RSL3, the classical ferroptosis inducers, widely inhibit tumor growth separately or synergistically with chemotherapy drugs in cancers, such as lung cancer, ovarian cancer, and pancreatic cancer (Li et al., 2020; Ye et al., 2020; Zhao et al., 2020; Ghoochani et al., 2021). However, the role of ferroptosis induction in CRC is controversial. Several CRC cell lines have been reported to be more resistant to ferroptosis induced by RSL3 or erastin than other cancer cells (Yang et al., 2014; Kraft et al., 2020). Therefore, several studies have been conducted to improve CRC cell sensitivity to ferroptosis induction.

Previous studies have demonstrated that glutathione peroxidase 4 (GPX4) acts as a central regulator in ferroptosis by inhibiting lipid peroxidation (Yang et al., 2014). Erastin and RSL3 trigger cell ferroptosis by blocking GPX4-mediated antioxidation (Yang and Stockwell, 2008; Xie et al., 2016; Yang and Stockwell, 2016; Stockwell et al., 2017). Currently, most studies have focused on the canonical GPX4 redox system (Xia et al., 2020; Wang et al., 2021a; Wang et al., 2021b; Ye et al., 2021). For example, serine and arginine-rich splicing factor 9 (SFRS9) was found as a target to improve CRC cell response to erastin through the regulation of GPX4 (Wang et al., 2021a). However, those studies and findings are limited to the GPX4 pathway and fail to explain why some CRC cells with low GPX4 levels are resistant to ferroptosis induction. The underlying regulatory mechanisms of CRC cell tolerance to ferroptosis induction remain elusive.

As widely known, many cancer cells can acquire massive endogenous antioxidant capacity for defense against cell death while under persistent oxidative stress (Trachootham et al., 2009). Recent studies have shown that another effective GPX4-independent antioxidative mechanism exists in cancer cells. GTP cyclohydrolase-1/tetrahydrobiopterin (GCH1/BH4) prevents cells from lipid peroxidation damage during ferroptosis induction; it is parallel to the GPX4 redox system (Kraft et al., 2020; Soula et al., 2020). BH4 is an essential cofactor and plays multiple roles in many physiological and pathological processes, including the regulation of oxidative stress and inflammation (Fanet et al., 2021). GCH1 is a critical enzyme for BH4 biosynthesis (Harada et al., 1993; Thöny et al., 2000). Some studies have reported that GCH1-mediated *de novo* synthesis of BH4 promotes CRC cell growth; and inhibition of GCH1 represses CRC progression *in vitro* and *in vivo* (Pickert et al., 2013). Nonetheless, the role of GCH1/BH4 metabolism in CRC ferroptosis is unknown. This study sought to investigate the function and underlying regulatory mechanism of GCH1/BH4 metabolism in CRC cells resistant to ferroptosis and demonstrate its potential implications in CRC therapy.

## Materials and Methods

### Cell Lines

The HCT116 cell line was purchased from the American Type Culture Collection (ATCC). The HT29 cell line was obtained from the Cell Bank of the Chinese Academy of Science (Shanghai, China). The SW480 and Caco-2 cell lines were stored by the Hubei Clinical Center and Key Laboratory of Intestinal and Colorectal Diseases (Zhongnan Hospital of Wuhan University). HCT116 was cultured in McCoy’s 5A. HT29 and SW480 were cultured in an RPMI-1640 medium. Caco-2 was cultured in a MEM medium. All cells were incubated in a 37°C incubator with 5% CO_2_ and maintained in a basic medium supplemented with 10% fetal bovine serum and 1% Penicillin/Streptomycin.

### Chemicals

Erastin (HY-15763), DAHP (HY-100954), 3-methyladenine (3-MA) (HY-19312), L-ascorbic acid (HY-B0166), and Bafilomycin A1 (HY-100558) were purchased from MedChemExpress. RSL3 (S8155) and ferrostatin-1 (S7243) were purchased from Selleck. BH4 (T4425) and 6-Biopterin (B2517) were purchased from Sigma Aldrich.

### Determination of BH4 Levels by HPLC

The oxidized species of BH4 and BH2 were measured using high-performance liquid chromatography (HPLC), as previously described (Zhang et al., 2019). Cellular lysates were extracted using lysis buffer (Beyotime Biotechnology, China), supplemented with 1% protease inhibitor cocktail, centrifuged at 17,000 × *g* at 4°C for 30 min, and then subjected to oxidation in acid and base. Under acidic conditions (1-N HCl containing 1% iodine and 2% potassium iodide), both BH2 and BH4 were oxidized to biopterin. However, only BH2 was oxidized to biopterin under basic conditions (1-N NaOH containing 1% iodine and 2% potassium iodide). Therefore, the difference between biopterin in acidic oxidation and basic oxidation determined the level of BH4 (Supplementary Figure S1). After being fully oxidized for an hour, all samples were vortexed and centrifuged at 17,000 × *g* for 30 min at 4°C. The supernatant (20 μl) was injected into an HPLC system with an autosampler and fluorescence detector (Agilent 1100). The C18 column (150 × 4.6 mm) was used to separate biopterin with a mobile phase of methanol (5%)/potassium phosphate buffer (95%) (50 mmol·L^−1^, pH = 3.0) running at a flow rate of 1.0 ml·min^−1^. The retention time of biopterin was approximately 6 min, and the λ excitation and emission were 350 and 440 nm, respectively. The compounds were quantified by their peak area in comparison with external standards (6-Biopterin) and normalized to protein concentration.

### Cell Viability Assay

Cells were seeded at 5,000 cells per well in 96-well plates. Twenty-four hours after seeding, the cells were transfected with siRNA, and 24 h later, erastin or RSL3 was added at different concentrations for 24 h. Ultimately, the cell viability was measured using cell counting Kit-8 (CCK-8) (Dojindo, Japan), according to the manufacturer’s instructions.

### Cell Death Assay

A total of 3 × 10^5^ cells were seeded into a 6-cm dish. After attachment overnight, cells were supplemented with varying concentrations of indicated compounds for 24 h. Cell death was measured with annexin V-FITC Apoptosis Detection Kit (BestBio, China), according to the manufacturer’s instructions.

### Lipid Peroxidation Assay

Cells were seeded in 6-well plates (3.0 × 10^5^ cells/well) and exposed to the indicated reagents. Afterwards, 2 μl of 10 mM BODIPY-C11 (D3861, Thermo Fisher Scientific) was added and incubated at 37°C for 30 min. All cells, including some detached and floating cells, were harvested and washed twice with PBS. Thereafter, the supernatant was separated, and the cell pellet was resuspended with 500 µl PBS. The signal of the oxidized C11 (FL1 channel) was then monitored, and the ratio of the FITC^+^ population relative to control cells was calculated using the FlowJo 10.4 software (Flow4, LLC). The mean percentages ±SEM of positive cell ratio were plotted.

### RNA Extraction and Quantitative Polymerase Chain Reaction

Total RNAs of siNC or siGCH1-transfected cells were isolated and extracted using TRIzol reagent (15596026, Invitrogen), and cDNA was synthesized using TOYOBO ReverTra Ace Kit (TOYOBO, Japan). The cDNA was then used as a template for the qRT-PCR experiments using UltraSYBR Mixture (CWbio, China). The primers were synthesized by TSINGKE Biological Technology (Wuhan, China). All qPCR data were normalized to the internal control gene expression of GAPDH to ensure accurate gene quantification. The genes and their respective parameters are listed in Supplementary Table S1.

### Western Blot Protein Analysis

Protein blotting was performed, as previously described (Xu et al., 2020). Briefly, cells were lysed with RIPA lysis buffer (Beyotime Biotechnology, China) containing 1% fresh protease inhibitor cocktail. Protein concentrations were quantified by BCA Protein assay kit (Beyotime, China). Cell lysates (30 μg protein/line) were separated by 12.5% SDS-PAGE and transferred onto nitrocellulose membranes (Millipore Corp, Billerica, MA, United States). The blotted membranes were blocked with Protein Free Rapid Block Buffer (EpiZyme, PS108) for 30 min and incubated with the corresponding primary antibodies at 4°C overnight. The primary antibodies against GCH1 (ab236387; Abcam; United States), SLC7A11 (ab175186; Abcam; United States), GPX4 (ab125066; Abcam; United States), FTH1 (#4393S; Cell Signaling Technology; United States), LC3B (#12741; Cell Signaling Technology; United States), NCOA4 (#66849; Cell Signaling Technology; United States), DMT1 (#15083; Cell Signaling Technology; United States), NRF2 (ab62352; Abcam; United States), TFR1 (#13113; Cell Signaling Technology; United States), xCT (ab62352; Abcam; United States), Beclin 1 (ab227107; Abcam; United States), HO-1 (A1346; Abclonal; China), and GAPDH (10494-1-AP; Proteintech; China). After washing the membranes with fresh TBST buffer, secondary antibodies of the peroxidase affinipure goat anti-rabbit or mouse IgG (1:5000; Promoter; China) were added and incubated for 2 h. Protein detection was performed based on the enhanced chemiluminescence (ECL) method and visualized using a Tanon-5200 Chemiluminescent Imaging System (Tanon Science and Technology, Shanghai, China). Protein bands were quantified using ImageJ.

### MDA Assay

The measurement of TBARS is a well-established method for monitoring malondialdehyde (MDA) content, a product of lipid peroxidation. Cells and tissues were assayed using a commercial kit (Cayman Chemicals 10009055), according to a modified version of the manufacturer’s protocol. Briefly, 100 μl cell lysates or tissue homogenates, 100 μl of SDS solution, and 4 ml of the color reagent were mixed in a 5 ml vial. Then, the vials were capped, boiled for 1 h, and incubated on ice immediately for 10 min. Following the ice incubation, the vails were centrifuged for 20 min at 1,800 × *g* at room temperature. The 1 ml of supernatant was transferred and loaded at 150 μl onto the black plate. The fluorescence was measured using PerkinElmer EnSpire (PE Enspire, Singapore) and λ excitation = 530 nm and λ emission = 550 nm.

### Gene Set Enrichment Analysis

The GSEA software and MSigDB gene sets were downloaded from the GSEA database (https://www.gsea-msigdb.org/gsea/index.jsp). The data of 471 patients with colon adenocarcinoma (COAD) were downloaded from The Cancer Genome Atlas (TCGA; www.cancergenome.nih.gov). Thereafter, GSEA was performed to further understand the biological function of GCH1 by analyzing Gene Ontology (GO) terms. The number of permutations was set to 1,000. The enrichment pathways with a *p*-value < 0.05 and enrichment score (ES) being correspondingly high were considered statistically significant.

### Cell Transfection

GCH1 siRNAs were synthesized by RiboBio (Guangzhou, China), and transiently transfected with cells using the Lipofectamine 2000 transfection reagent (Invitrogen, Green Island, CA). After 4–6 h, the medium was refreshed with a complete medium, and cells were cultivated overnight to prepare the following treatment. The GCH1 siRNA sequence data are listed in Supplementary Table. S2.

### Cellular and Mitochondrial Iron Detection

The iron concentration was assessed by using FerroOrange (Dojindo, F374, Japan) and Mito-FerroGreen probes (Dojindo, M489, Japan), according to the manufacturer’s protocol. Treated cells were washed twice with Hank’s Balanced Salt Solution (HBSS) and later fixed with a final concentration of 5 μM FerroOrange or 5 μM Mito-FerroGreen diluted with HBSS. Thereafter, the cells were incubated in the dark at 37°C for 30 min. The FerroOrange-stained cells were directly incubated with Hoechst 33342 (Solarbio, C0031, China) (5 μg/ml) without washing, followed by confocal microscopy detection. The Mito-FerroGreen-stained cells were washed twice with HBSS, incubated with Hoechst 33342 (5 μg/ml) for 5 min, washed with HBSS again, and finally observed under a confocal microscope. ImageJ was used to determine the fluorescence intensity.

### Xenograft Mouse Model

Male athymic nude mice (3 weeks old) were purchased from GemPharmatech (Jiangsu, China). The experiments were conducted under a protocol approved by the Zhongnan Hospital of Wuhan University Institutional Animal Care Animal Welfare Committee. HCT116 cells (3 × 10^6^ cells/0.1 ml) were implanted subcutaneously in the right side of the dorsal midline of the mice. When the tumor volume reached 80–180 mm^3^, the mice were assigned randomly into different treatment groups and treated with vehicle (i.p., once every other day), DAHP (80 mg/kg, i.p., once every other day), erastin (40 mg/kg, i.p., once every 2 days), and combination treatment (DAHP plus erastin) for 10 days. All the drugs were dissolved in 5% DMSO +40% PEG + 5% Tween 80 + 50% physiological saline. The solution was sonicated to aid dissolution. The mice were sacrificed 11 days after drug administration. Tumors were measured every 2 days until the endpoint; the tumor volume was calculated using the following formula: length × width^2^ × 1/2.

### Histology and Immunohistochemistry

The freshly isolated tumor tissues were fixed in 4% paraformaldehyde overnight, embedded in paraffin, sectioned, and mounted on slides. Hematoxylin and eosin (H&E) staining or immunohistochemistry (IHC) was performed using routine methods. In brief, the primary antibodies, including GCH1 (1:500, ab236387, Abcam), Ki-67 (1:500, 9027s, Cell Signaling Technology), Act-caspase 3 (1:500, 9661s, Cell Signaling Technology), and 4-HNE (1:400, ab46545, Abcam), were incubated at 4°C overnight. Staining was performed using the Vectastain elite ABC kit and DAB peroxidase substrate kit (Vector Laboratories). Images were randomly taken from five samples per group at ×200 magnification using an Olympus BX43 microscope. Afterwards, the image analysis was performed using IHC Profiler of ImageJ, which calculates the percentage of positive cells automatically (Varghese et al., 2014; Jia et al., 2021).

### Statistical Analysis

Data were presented as mean ±SEM. All experiments were performed independently in triplicate. Statistical significance was determined using two-way analysis of variance (ANOVA) or two-tailed Student’s *t*-test. GraphPad *Prism 8.0* software was used to analyze and visualize data. *p* < 0.05 was considered to be statistically significant.

## Results

### GCH1/BH4 Metabolism Negatively Correlates With CRC Cell Sensitivity to Erastin-Induced Ferroptosis

**FIGURE 1:**
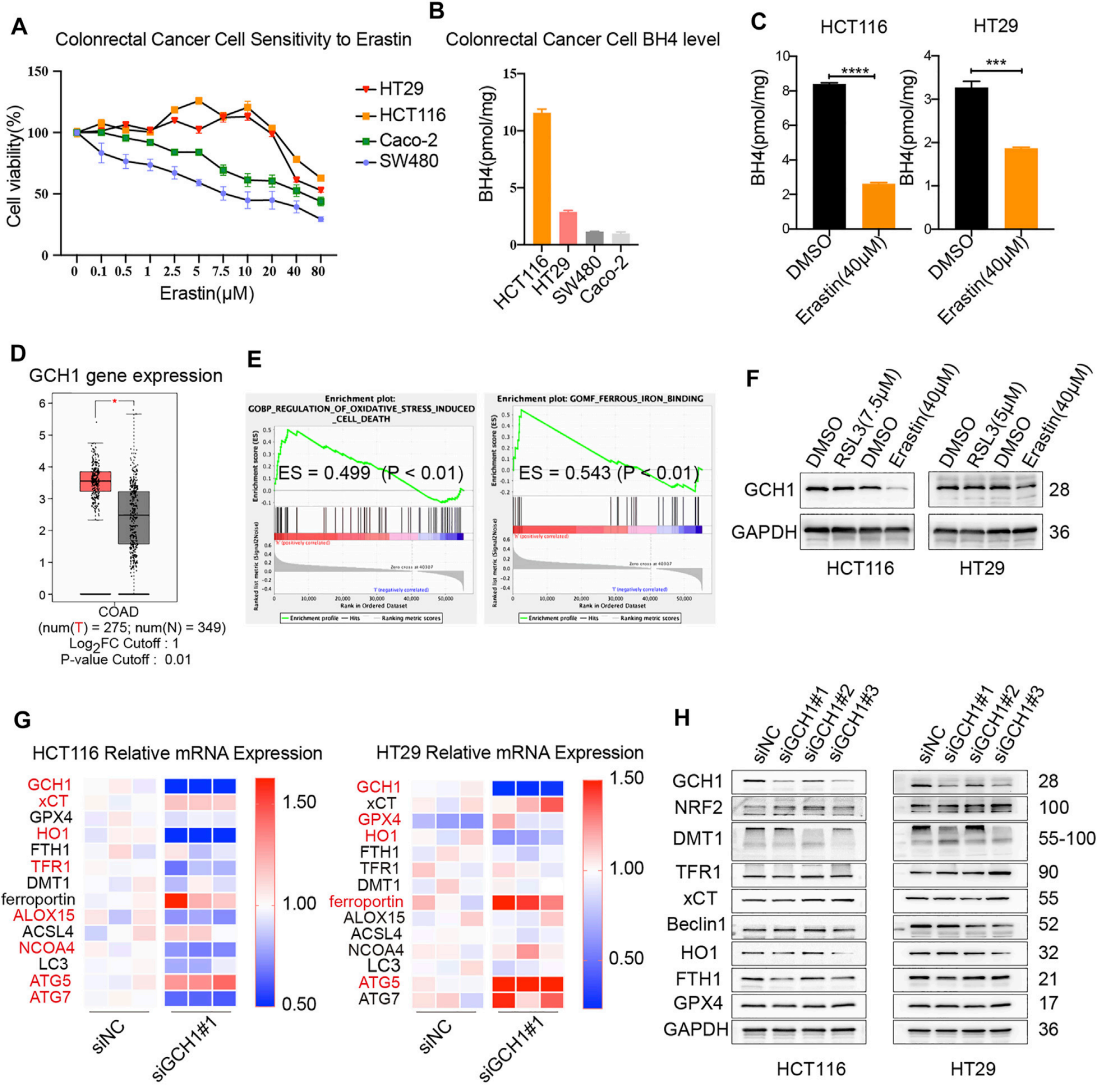
Association between GCH1/BH4 metabolism and CRC ferroptosis. **(A–C)** The correlation between BH4 and erastin-induced cell death. **(A)** Cell viability upon treatment with different doses of erastin for 24 h in CRC cell lines. **(B)** BH4 abundance across 4 CRC cell lines at baseline. **(C)** BH4 levels were measured in HCT116 and HT29 cells treated with erastin for 24 h. The error bars represent standard deviation from three replicates (***p < 0.001, ****p < 0.0001, compared between the two groups by unpaired *t*-test). **(D**–**F)** Association between GCH1 and ferroptosis in CRC. **(D)** GCH1 expression of colon adenocarcinoma (COAD) vs normal adjacent tissues in Gene Expression Profiling Interactive Analysis tool (GEPIA) *p < 0.05. **(E)** GSEA showing the regulation of oxidative stress-induced cell death and ferrous iron binding pathways with apparent enrichment in low or high expression of GCH1 among patients with CRC. ES, enrichment score. **(F)** GCH1 protein level was measured in HCT116 and HT29 cells treated with RSL3 or erastin for 24 h. **(G‒H)**, mRNA **(G)** and protein **(H)** expression of ferroptosis-related genes in the transfected cells. **(G)** Red color marked molecules with statistical difference (*p* < 0.05) between siNC and siGCH1 transfected cells.

The analysis of the association between GCH1/BH4 metabolism and CRC cell ferroptosis was conducted in four human CRC cell lines, including HCT116, HT29, SW480, and Caco-2 cells. Cell sensitivity to ferroptosis and its corresponding BH4 content were assessed. Interestingly, HCT116 and HT29 cells harboring elevated levels of BH4 were resistant to erastin-induced cell death (Figures 1A,B). However, most of the cell lines were resistant to RSL3-induced cell death, regardless of BH4 levels (data not shown). This result shows that BH4 is negatively associated with CRC cell sensitivity to erastin-induced ferroptosis. Thereafter, all the following *in vitro* experiments of this study were conducted using HCT116 and HT29 cells.

To explore the effect of ferroptosis inducers on BH4, the level of BH4 was detected in cells treated with erastin or RSL3. As shown in Figure 1C, BH4 was markedly decreased in cells after erastin treatment. However, no change of BH4 upon RSL3 induction was observed (data not shown). Therefore, BH4 is closely related with erastin rather than RSL3 induction. BH4 seems more likely to participate in erastin-induced metabolic changes and serves as a pharmacodynamic marker of erastin.

To further investigate the role of GCH1 in ferroptosis, data on patients with CRC were collected from TCGA cohort, and the level of GCH1 mRNA was analyzed. GCH1 mRNA was significantly higher in CRC than in normal tissues (Figure 1D). GSEA revealed that GCH1 was closely correlated with the regulation of oxidative stress-induced cell death and ferrous iron binding (Figure 1E), suggesting that GCH1 is correlated with iron-dependent ferroptosis in CRC cells. Consistent with the change of BH4, GCH1 expression was repressed after erastin induction rather than RSL3 (Figure 1F). Those findings indicate that GCH1/BH4 metabolism participates in erastin-induced ferroptosis.

To verify the effect of GCH1/BH4 metabolism in ferroptosis, small interfering RNA (siRNA) specific to GCH1 was applied. GCH1 siRNA 1 and 3 markedly blocked GCH1 expression, and siRNA 1 (briefly called siGCH1) was used for the following GCH1 siRNA knockdown experiments. The mRNAs and proteins of ferroptosis-related genes were assayed after GCH1 siRNA treatment (Figures 1G‒H). Heme oxygenase 1 (HO-1), an important antioxidant response enzyme and free iron regulator, drastically reduced both mRNA and protein levels upon GCH1 knockdown in CRC cells (Grochot-Przeczek et al., 2012). The transcription factor, nuclear factor erythroid 2-related factor 2 (NRF2), exquisitely modulates ferroptosis by targeting a host of ferroptosis cascade genes (Lu et al., 2021). Knockdown of GCH1 moderately increased the protein levels of NRF2. In addition, autophagy-related genes (ATG), including ATG5 and ATG7, are required machinery of ferroptosis for initiating oxidative injury (Liu et al., 2019). The mRNA levels of ATG5 were consistently upregulated in both HCT116 and HT29 cells upon GCH1 knockdown. Besides, GCH1 siRNA significantly altered the expression of some iron transport proteins, in which divalent metal transporter 1 (DMT1) was downregulated and transferrin receptor 1 (TFR1) was upregulated. Remarkably, ferritin (FTH1), the main ferrous iron storage protein complex (Harrison and Arosio, 1996), was downregulated on protein levels in both HCT116 and HT29 cells after GCH1 silencing. However, the classic ferroptosis antioxidation molecules of SLC7A11 (xCT) and GPX4 were unchanged. Taken together, the results above support that GCH1/BH4 metabolism participates in erastin-induced ferroptosis and acts extensively on the antioxidant and iron-binding system independent of the classic ferroptosis antioxidation pathway.

### Deletion of GCH1/BH4 Metabolism Mitigates Resistance to Erastin-Induced Ferroptosis Rather Than RSL3 in CRC Cells

**FIGURE 2:**
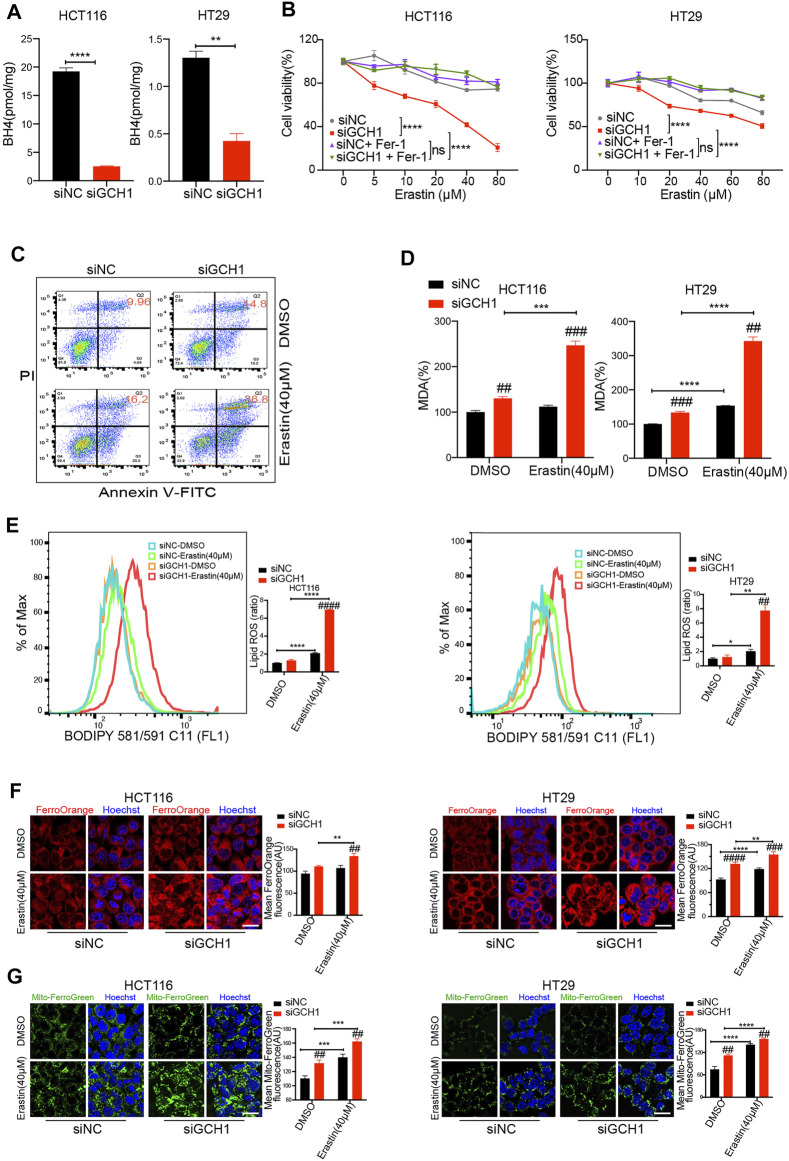
Genetic deletion of GCH1/BH4 metabolism and induction of erastin synergistically induce ferroptosis in CRC cells. **(A)** BH4 levels were largely decreased by GCH1 knockdown for 24 h. **(B**,**C)** GCH1 knockdown sensitized HCT116 and HT29 cells to erastin. **(B)** Cell viability in HCT116 and HT29 cells treated with different doses of erastin and co-treated with 2 µM ferrostatin-1 for 24 h (****p < 0.0001, ns, not significant, the total variation between the two groups examined by two-way ANOVA with Tukey’s post hoc test). **(C)** Representative FACS plots of annexin V-FITC/PI staining in HCT116 cells. **(D‒E)**, GCH1 knockdown promoted erastin-induced lipid peroxidation, measured using the MDA assay kit **(D)** or BODIPY-C11 staining **(E)** in HCT116 and HT29 cells (Bar graph showing the ratio of lipid ROS normalized to DMSO-treated siNC cells). **(F**,**G)** GCH1 knockdown promoted erastin-induced cellular Fe^2+^ **(F)** and mitochondrial Fe^2+^ **(G)** accumulation, detected separately using FerroOrange and MitoferroGreen staining. Scale bar, 20 µM. The error bars represent standard deviation from at least three replicates (##*p* < 0.01, ###*p* < 0.001, ####*p* < 0.0001, compared between siNC and siGCH1 by unpaired *t*-test) (*p < 0.05, **p < 0.01, ***p < 0.001, ****p < 0.0001, compared between the two groups by unpaired *t*-test).

To investigate the exact role of GCH1/BH4 in ferroptosis resistance, ferroptosis was assayed after GCH1 knockdown. The BH4 levels were downregulated in GCH1 silencing cells (Figure 2A). Notably, the decline of BH4 was more remarkable in HCT116, which had a higher level of BH4 than HT29 cells. First, the descent of cell viability was measured by CCK-8. Cell death was investigated by annexin V-FITC/PI dual staining and visualized under microscopes. Cells with GCH1-knockdown were more susceptible to erastin treatment than control cells (Figures 2B,C and Supplementary Figure S2). Importantly, as shown in Figure 2B, erastin-induced cell death was suppressed by the ferroptosis inhibitor, ferrostatin-1. Those results indicate that GCH1/BH4 deletion promotes erastin-induced ferroptosis in CRC cells.

Second, the effect of GCH1/BH4 deletion on lipid peroxidation was detected. Lipid peroxidation is one feature of ferroptosis (Li and Li, 2020). The level of lipid peroxidation in the cells was indexed by malondialdehyde (MDA) level and BODIPY581/591 staining. After erastin treatment, lipid peroxidation was significantly increased in GCH1 silencing cells, compared to the controls (Figures 2D,E). These results indicate that GCH1/BH4 deletion tremendously enhances lipid peroxidation induced by erastin in CRC cells.

Third, ferrous iron (Fe^2+^) accumulation was examined in the cells after GCH1/BH4 inhibition. Fe^2+^ accumulation is another feature of ferroptosis, which causes excessive generation of reactive oxygen species (ROS) through the Fenton reaction (Dixon et al., 2012). The results revealed that both intracellular Fe^2+^ levels and mitochondrial Fe^2+^ levels increased significantly in GCH1 silencing cells in response to erastin treatment, compared to control cells (Figures 2F,G). These findings indicate that GCH1/BH4 deletion enhances Fe^2+^ accumulation upon erastin stimulation in CRC cells.

In addition, to validate whether GCH1/BH4 deletion could enhance RSL3 pharmacodynamic effects, we assessed ferroptosis during RSL3 treatment. Notably, GCH1 silencing failed to promote RLS3-induced cell death (Supplementary Figure S3A) and lipid peroxidation (Supplementary Figures S3B,C). These results demonstrate that GCH1/BH4 deletion can enhance erastin susceptibility but cannot overcome RSL3 resistance in CRC.

### Supplementation With BH4 Eliminates the Effect of GCH1 Knockdown on Erastin-Induced Ferroptosis in CRC Cells

**FIGURE 3:**
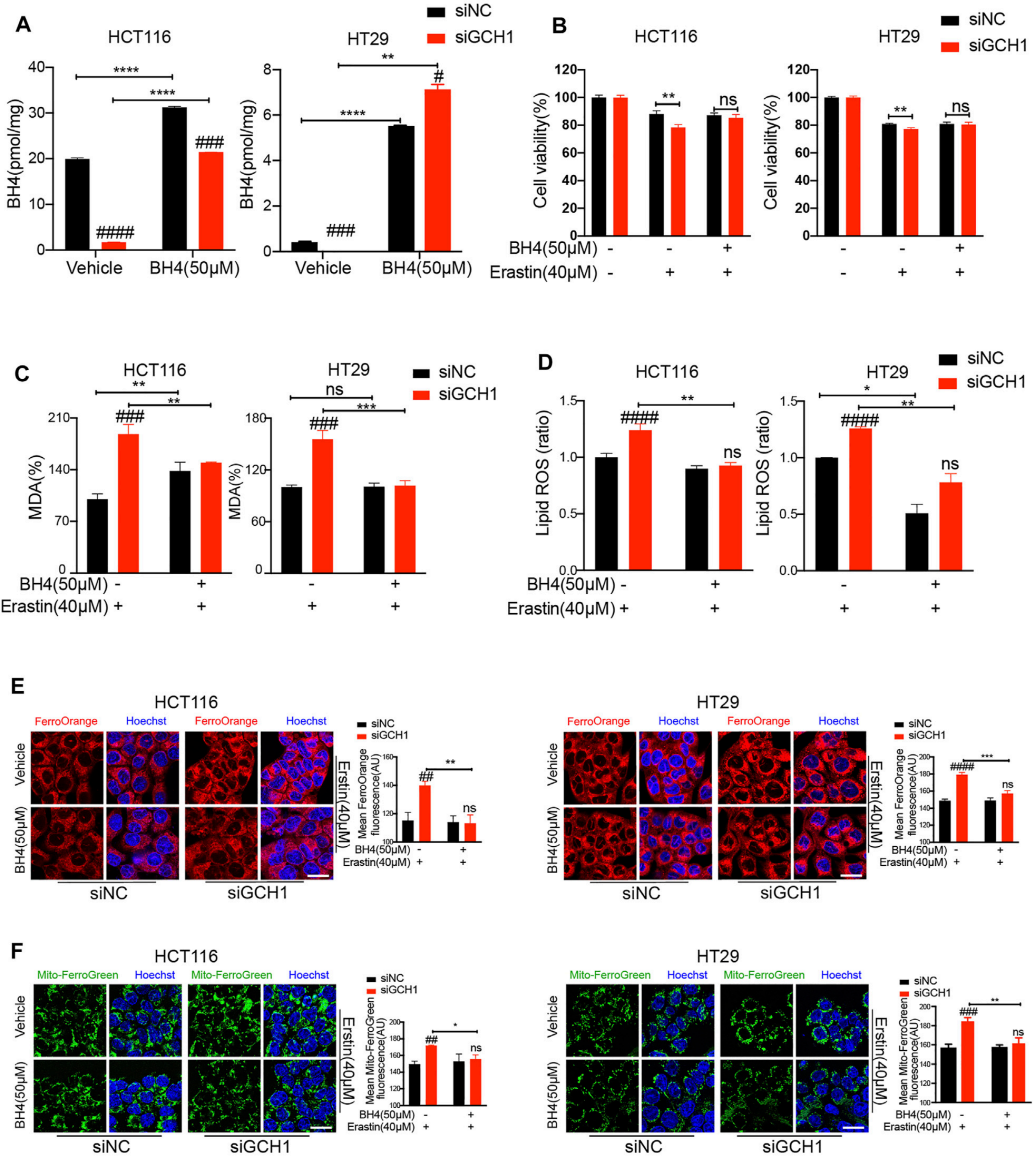
Supplementation of BH4 protects CRC cells from ferroptosis promoted by GCH1 knockdown. **(A)** Supplementation of 50 µM BH4 restored the BH4 levels in siGCH1 transfected cells. **(B**-**F)** BH4 supplemented in **(A)** reversed cell death, lipid peroxidation, and ferrous iron accumulation caused by GCH1 knockdown during erastin treatment. **(B)** Cell viability was assessed by CCK-8. Lipid peroxidation was detected using the MDA assay kit **(C)** or BODIPY-C11 staining **(D)** (The ratio of lipid ROS normalized to erastin-treated siNC cells). The cellular Fe^2+^ **(E)** and mitochondrial Fe^2+^ **(F)** were detected using FerroOrange and MitoferroGreen staining, separately. Scale bar, 20 µM. The error bars represent standard deviation from at least three replicates (#*p* < 0.05, ##*p* < 0.01, ###*p* < 0.001, ####*p* < 0.0001, ns, not significant, compared between siNC and siGCH1 by unpaired *t*-test) (*p < 0.05, **p < 0.01, ***p < 0.001, ****p < 0.0001, ns, not significant, compared between the two groups by unpaired *t*-test).

Considering that BH4 is a critical regulator of redox homeostasis (Kim et al., 2007), we reasoned that GCH1 deletion reversed erastin resistance through BH4. Therefore, we examined whether the effect of GCH1-knockdown on erastin-induced ferroptosis would be abrogated under BH4 supplementation. After BH4 exogenous supplementation, the BH4 content of GCH1-knockdown cells was proximately restored to normal levels in control cells (Figure 3A). Moreover, BH4 administration reversed erastin-induced cell death (Figure 3B), lipid peroxidation production (Figures 3C,D), and Fe^2+^ accumulation (Figures 3E,F) in GCH1-knockdown CRC cells. These findings support the hypothesis that GCH1 knockdown elevates erastin sensitivity by blocking BH4 biosynthesis.

### Blockade of GCH1/BH4 Selectively Promotes Erastin-Induced Ferroptosis by Activating Ferritinophagy

**FIGURE 4:**
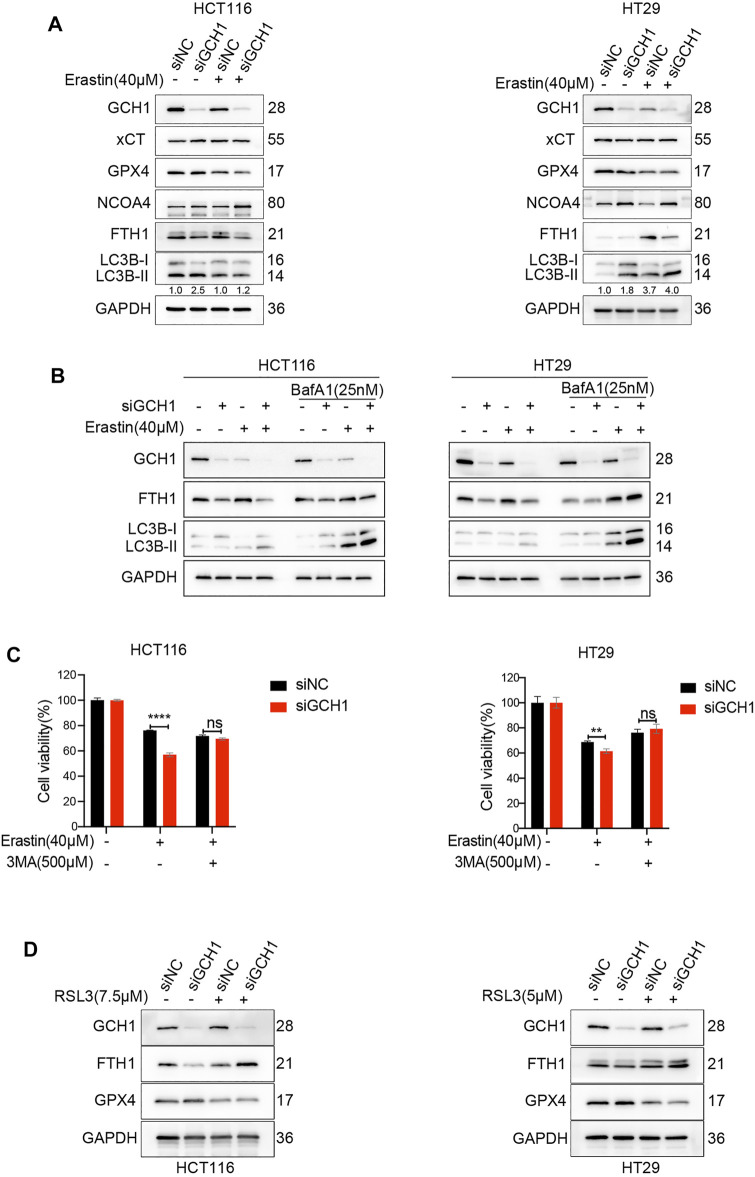
Blockade of GCH1/BH4 sensitizes erastin-induced ferroptosis by activating ferritinophagy. **(A)** Western blot analysis of GCH1, xCT, GPX4, NCOA4, FTH1, LC3B, and GAPDH in HCT116 and HT29 cells treated with 40 µM erastin for 24 h. The numbers below the LC3 lane indicate the ratio of LC3B-II/LC3B-I. **(B)** Autophagic flux was determined by the accumulation of LC3B-II in a 4-h treatment period with 25 nM bafilomycin A1 (BafA1). **(C)** Cell viability after pretreatment with 3-methyladenine (3-MA) for 24 h and treatment with erastin for 24 h in HCT116 and HT29 cells. **(D)** Western blot analysis of GCH1, FTH1, GPX4, and GAPDH in HCT116 and HT29 cells treated with RSL3 for 24 h. The error bars represent standard deviation from at least three replicates (**p < 0.01, ****p < 0.0001, ns, not significant, compared between the two groups by unpaired *t*-test).

We subsequently explored the underlying mechanism by which GCH1/BH4 regulates CRC ferroptosis. We noticed that BH4 deficiency sensitized CRC cells to erastin rather than RSL3 treatment and drastically enhanced free iron accumulation during erastin treatment (Supplementary Figure S3). Additionally, knockdown of GCH1 suppressed FTH1 protein levels (Figure 1H). Previous studies demonstrated that nuclear receptor coactivator 4 (NCOA4)-mediated selective autophagic turnover of FTH1 (namely ferritinophagy) is necessary for erastin but unnecessary for RSL3 (Liu et al., 2020; Mancias et al., 2014; Gryzik et al., 2021). To investigate whether GCH1/BH4 deficiency selectively promotes erastin-induced ferroptosis through activation of ferritinophagy, the NCOA4, FTH1, and LC3B protein levels were assessed. As shown in Figure 4A, GCH1-knockdown increased LC3B-II/LC3B-I ratio and NCOA4 protein level, indicating selective ferritinophagy activation (Zhang et al., 2021). Additionally, GCH1-knockdown drastically decreased FTH1 protein level during erastin treatment, which was a consequence of ferritinophagy. Moreover, we evaluated the efficient autophagic flux through western blot of LC3B-II in the presence or absence of bafilomycin A1 (BafA1), an inhibitor of the fusion of autophagosomes and lysosomes (Barth et al., 2010). GCH1-knockdown exacerbated the accumulation of LC3B-II induced by BafA1 after erastin treatment, which indicated remarkable activation of autophagic flux. FTH1 level decreased during autophagy activation, which could be reversed slightly by the transient use of BafA1 (Figure 4B). However, as shown in Figure 4A, GPX4 and xCT protein levels were maintained during GCH1-knockdown. Therefore, GCH1/BH4 metabolism, independent of the glutathione or cystine transport system, causes ferroptosis resistance through NCOA4-mediated ferritinophagy in CRC.

To confirm the essential role of ferritinophagy activation in GCH1/BH4 deficiency-enhanced erastin-induced ferroptosis, the cell lines were treated with 3-methyladenine (3MA), which inhibits the conversion of LC3B-I to LC3B-II. The results showed that 3MA significantly reversed cell death promoted by GCH1/BH4 deficiency during erastin treatment (Figure 4C).

To explore whether ferritinophagy activation was involved in RSL3-induced ferroptosis, we assessed FTH1 protein during RSL3 treatment. Although knockdown of GCH1 decreased FTH1 level during control of DMSO treatment, it failed to decrease FTH1 level during RSL3 treatment (Figure 4D). Hence, these results demonstrate that GCH1/BH4 deficiency enhances NCOA4 expression and ferritinophagy activation, which fuels cells with more free irons and remarkably accelerates cell death induced by erastin rather than RSL3.

### Pharmacological Inhibition of GCH1/BH4 Metabolism Sensitizes CRC Cells to Erastin-Induced Ferroptosis *In Vitro* and *In Vivo*

2,4-diamina-6-hydroxypyrimidine (DAHP) is a specific inhibitor of GCH1 (Kolinsky and Gross, 2004; Ceylan-Isik et al., 2009). To examine the possibility that drug inhibition of GCH1 could be a clinically relevant approach to sensitize CRC to erastin-induced ferroptosis, we performed DAHP treatment *in vitro* and *in vivo*. First, to exclude drug toxicity, we investigated the safety profile of DAHP, which revealed that using DAHP at a concentration of ≤1 mM was harmless for CRC cells (Supplementary Figure S4A). Therefore, DAHP with a concentration of 500 µM was used for the following *in vitro* experiments. As expected, BH4 was almost entirely inhibited by DAHP administration (Supplementary Figure S4B). Consistently, compared with single-drug treatment, cells co-treated with DAHP and erastin underwent excessive cell death (Supplementary Figure S4C), lipid peroxidation production (Supplementary Figures S4D,E), and free iron accumulation (Supplementary Figures S4F,G), which are characteristics of ferroptosis. Together, these findings suggest that pharmacologically inhibiting GCH1 with DAHP promotes erastin-induced ferroptosis *in vitro*.

**FIGURE 5:**
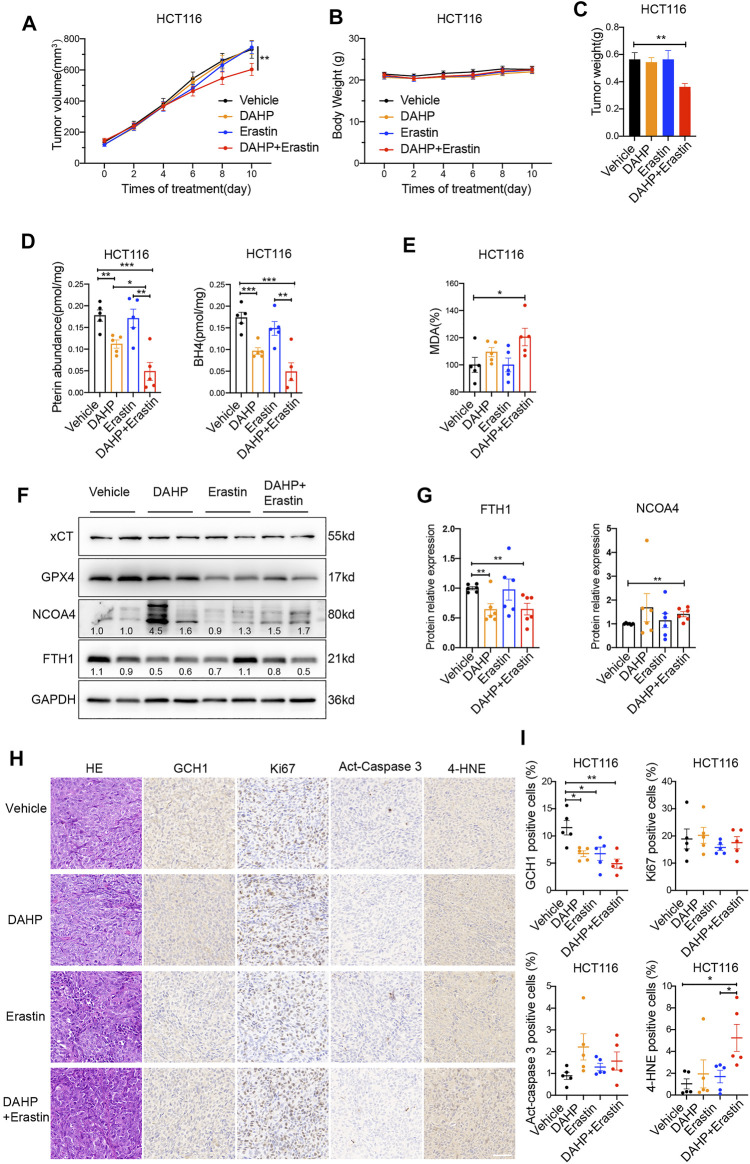
Co-treatment of DAHP and erastin suppresses tumor growth *in vivo*. **(A**,**B)** The tumor volume **(A)** and body weight **(B)** of HCT116 xenograft tumors with the indicated treatment (n = 8 mice/group, two-way ANOVA). **(C)** Weight of isolated tumors at day 11 (n = 8 mice/group, unpaired *t*-test). **(D**,**E)** In parallel, total pterin, BH4 **(D)**, and MDA levels **(E)** in the isolated tumors were assayed (n = 5 mice/group, unpaired *t*-test). **(F,G)** Representative western blot images **(F)** and quantitative analysis results **(G)** of indicated proteins in isolated tumors (n = 6 mice/group, unpaired *t*-test). **(H)** Representative H&E and IHC staining graphs of isolated tumors with indicated treatment. Scale bars, 50 μm. GCH1, Ki67, Act-caspase 3 and 4-HNE **(I)** positive cells shown in **(H)** were quantified using ImageJ (n = 5 mice/group, unpaired *t*-test). *p < 0.05, **p < 0.01, ***p < 0.001.

**FIGURE 6:**
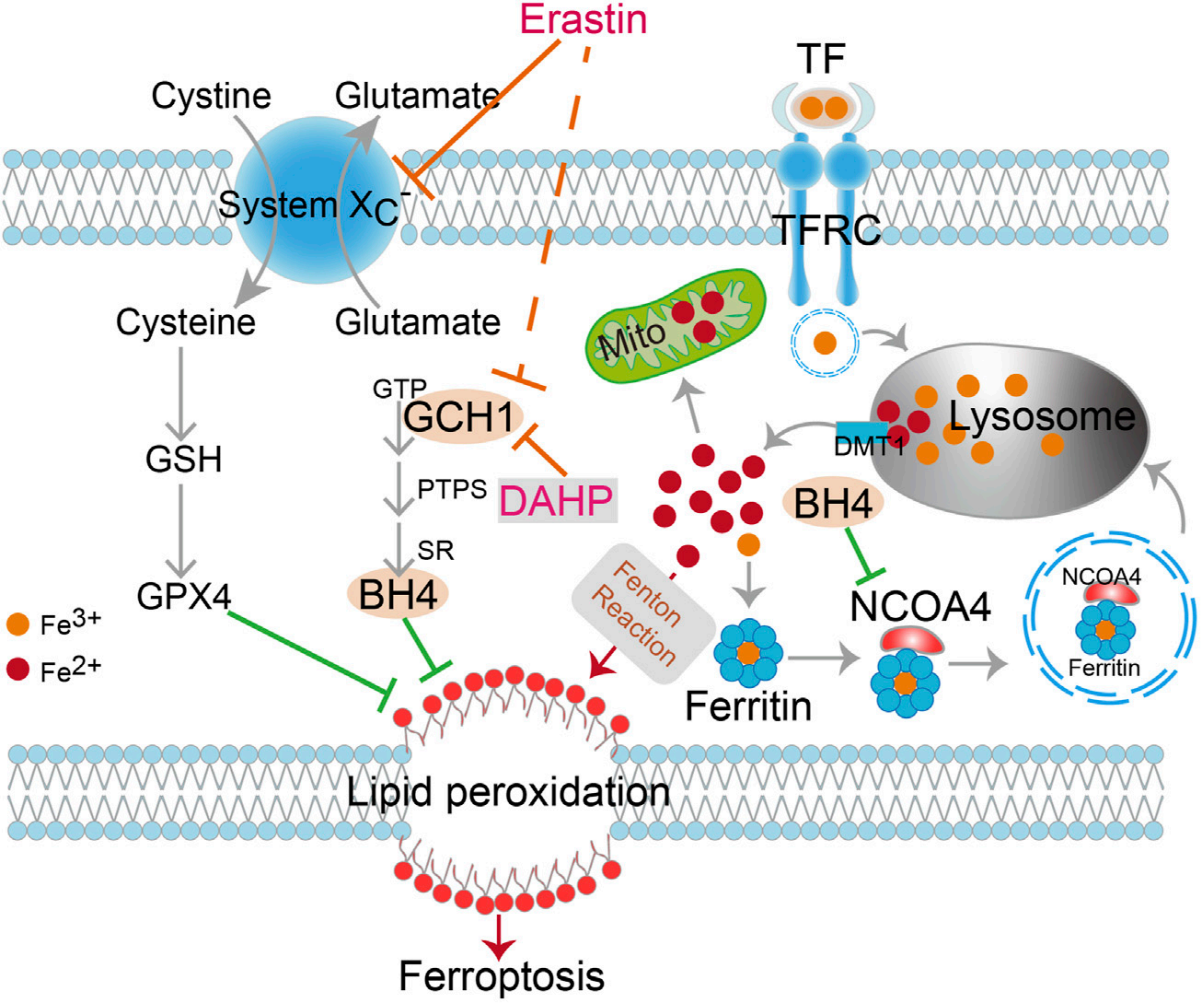
Schematic description of GCH1/BH4 metabolism suppression of erastin-induced ferroptosis by reducing lipid peroxidation and silencing NCOA4-mediated ferritinophagy in CRC. DAHP, an inhibitor of GCH1; TFR1, transferrin receptor 1; TF, transferrin; DMT1, divalent metal transporter 1; GSH, Glutathione; GPX4, Glutathione peroxidase 4; GCH1, GTP cyclohydrolase-1; BH4, tetrahydrobiopterin; PTPS, 6-pyruvoyl tetrahydropterin synthase; SR, sepiapterin reductase; Mito, mitochondria.

Furthermore, a xenograft tumor model was used to evaluate the anti-tumor activity of erastin combined with DAHP *in vivo*. In contrast to single-drug therapy, co-treatment with DAHP and erastin significantly suppressed the growth of the HCT116 xenografts without significant weight loss (Figures 5A–C). Based on the observation of the cell culture data *in vitro*, we investigated whether DAHP and erastin synergistically induced ferroptosis *in vivo*. Indeed, BH4, total pterin, and GCH1 proteins were significantly downregulated by DAHP alone, and this downregulation was further enhanced by the combination with erastin (Figures 5D,H,I). Besides, co-treatment with DAHP and erastin synergistically enhanced lipid peroxidation levels, as measured by the intra-tumoral MDA assay or 4HNE staining (Figures 5E,H,I). FTH1 was downregulated, and NCOA4 was enhanced by DAHP alone or in combination with erastin, indicating ferritinophagy activation (Figures 5F,G). DAHP administration had no effect on GPX4 or xCT protein levels, compared with control cells (Figure 5F). Notably, the combination of DAHP and erastin did not affect cell proliferation as measured by Ki67 or apoptosis induction as measured by act-caspase 3 (Figures 5H,I). These animal studies confirm that GCH1/BH4 metabolism limits the anticancer activity of erastin by suppressing lipid peroxidation and ferrous iron release in CRC. Additionally, GCH1/BH4 is implicated as a critical target for reversing ferroptosis resistance during erastin therapy (Figure 6).

## Discussion

Induction of ferroptosis is a potential therapeutic strategy for tumor eradication (Hassannia et al., 2019; Chen et al., 2021). However, the underlying mechanisms of susceptibility and resistance to ferroptosis, particularly in CRC, are still unclear. Elucidating the ferroptotic peculiarity in CRC and its underlying mechanisms is critical for developing personalized therapeutic strategies based on ferroptosis induction. Our results demonstrated that GCH1/BH4 metabolism is a novel ferroptosis-resistant mechanism in CRC, entirely independent of the GPX4 redox system. Blockade of GCH1/BH4 metabolism genetically or pharmacologically successfully promoted erastin-induced ferroptosis in CRC.

Ferrous iron plays a critical role in the occurrence of ferroptosis (Stoyanovsky et al., 2019). The homeostasis of iron is maintained by its import, export, and storage. Many studies reported that CRC cells are iron-rich and iron-dependent (Brookes et al., 2006; Chua et al., 2010; Radulescu et al., 2012). Interestingly, despite elevated levels of iron, CRC cells are still less susceptible to ferroptosis inducers than other cancers. Most irons are bound by ferritin, thereby preventing the production of the Fenton reaction (Torti and Torti, 2013). As a result, iron overload is not equivalent to massive availability of iron. The delicate balance between “free” iron availability and ferritin level seems to control ferroptosis. Importantly, our study revealed that silencing GCH1could break this equilibrium to restore the sensitivity of ferroptosis in CRC. We confirmed that NCOA4 level increased drastically during GCH1 inhibition and erastin treatment *in vitro* and *in vivo*, accompanied by FTH1 degradation and lipid peroxidation production. NCOA4 selectively recognizes and degrades FTH1 using autolysosomes (Gao et al., 2016; Hou et al., 2016). Meanwhile, the LC3B-II/LC3B-I ratio and autophagy flux were drastically increased in GCH1 knockdown cells, compared to the controls, during erastin treatment. To further confirm the essential role of ferritinophagy in GCH1 silencing-promoted ferroptosis, 3MA was applied to inhibit autophagy. Consistently, 3MA administration successfully reversed the part of cell death increased by knockdown of GCH1 during erastin treatment. Therefore, we firstly confirmed that the inhibition of GCH1/BH4 promotes ferroptosis by activating ferritinophagy. However, some iron transport proteins, including TFR1 and DMT1, were altered upon GCH1 knockdown. The role played by these iron transport proteins in this process remains elusive.

Two recent studies revealed that GCH1/BH4 metabolism possesses an antagonistic effect against ferroptosis as an endogenously antioxidant system in fibroblast cells, fibrosarcoma cells, leukemia cells, and pancreatic cancer cells (Kraft et al., 2020; Soula et al., 2020). However, the intervention of GCH1/BH4 to diverse ferroptosis inducers was discordant in both studies. Different from prior studies, our study indicated that GCH1/BH4 deletion selectively promoted erastin-induced cell death rather than RSL3 in CRC, due to selective ferritinophagy activation. The different genetic backgrounds of CRC, compared to fibrosarcoma and leukemia, may contribute to the differences. By checking their genetic profiles, we found that PIK3CA, which encodes the catalytic subunit of phosphatidylinositol 3-kinase (PI3K), was mutant in HCT116 and HT29 cells but not in others. Moreover, PI3K was reported to promote and regulate various cellular processes, and the inhibitor of PI3K was found to prevent erastin-induced ferroptosis (Kang et al., 2014). Therefore, a missense mutation of PIK3CA leading to the ferroptosis resistance in HCT116 and HT29 cells may be an extreme possibility. Further studies are needed to confirm this hypothesis.

Interestingly, the combination of DAHP and erastin significantly inhibited tumor growth but failed to affect tumor cell proliferation *in vivo*. Indeed, tumor growth is not only a consequence of uncontrolled proliferation but also a result of reduced cell death. The balance between proliferation and cell death is crucial in determining the growth of the tumor. Our data suggested that ferroptosis rather than disturbance of cell progression contributes to the suppression of tumor growth upon DAHP and erastin co-treatment. A similar finding has been reported in another study on ferroptosis (Mao et al., 2021). However, the exact interaction between proliferation and ferroptosis remains unclear.

Additionally, our findings demonstrated that GCH1 and downstream BH4 levels are significantly decreased by erastin induction in CRC. Although the exact mechanisms remain unknown, evidence suggests that erastin induction downregulates the translocation of nuclear factor erythroid 2-related factor 2 (NRF2) within the nucleus in lung cancer and GCH1 is one of the downstream genes of NRF2 in skin cells (Xue et al., 2017; Gai et al., 2020). Whether erastin decreases GCH1/BH4 metabolism through NRF2 in CRC merits further investigation.

In conclusion, this study revealed that GCH1/BH4 metabolism acts to inhibit erastin-induced ferroptosis by restraining NCOA4-mediated ferritinophagy, providing a potential antitumor target for amplifying the ferroptotic activity of erastin induction in CRC.

## Data Availability

The original contributions presented in the study are included in the article/Supplementary Material, further inquiries can be directed to the corresponding authors.

## References

[B1] Arnold M. Sierra M. S. Laversanne M. Soerjomataram I. Jemal A. Bray F. (2017). Global Patterns and Trends in Colorectal Cancer Incidence and Mortality. Gut 66 (4), 683–691. 10.1136/gutjnl-2015-310912 26818619

[B2] Barth S. Glick D. Macleod K. F. (2010). Autophagy: Assays and Artifacts. J. Pathol. 221 (2), 117–124. 10.1002/path.2694 20225337 PMC2989884

[B3] Benson A. B. Venook A. P. Al-Hawary M. M. Cederquist L. Chen Y.-J. Ciombor K. K. (2018). NCCN Guidelines Insights: Colon Cancer, Version 2.2018. J. Natl. Compr. Canc Netw. 16 (4), 359–369. 10.6004/jnccn.2018.0021 29632055 PMC10184502

[B4] Bray F. Ferlay J. Soerjomataram I. Siegel R. L. Torre L. A. Jemal A. (2018). Global Cancer Statistics 2018: GLOBOCAN Estimates of Incidence and Mortality Worldwide for 36 Cancers in 185 Countries. CA: A Cancer J. Clinicians 68 (6), 394–424. 10.3322/caac.21492 30207593

[B5] Brookes M. J. Hughes S. Turner F. E. Reynolds G. Sharma N. Ismail T. (2006). Modulation of Iron Transport Proteins in Human Colorectal Carcinogenesis. Gut 55 (10), 1449–1460. 10.1136/gut.2006.094060 16641131 PMC1856421

[B6] Ceylan-Isik A. F. Guo K. K. Carlson E. C. Privratsky J. R. Liao S.-J. Cai L. (2009). Metallothionein Abrogates GTP Cyclohydrolase I Inhibition-Induced Cardiac Contractile and Morphological Defects. Hypertension 53 (6), 1023–1031. 10.1161/hypertensionaha.108.123422 19398661 PMC2782760

[B7] Chen X. Kang R. Kroemer G. Tang D. (2021). Broadening Horizons: the Role of Ferroptosis in Cancer. Nat. Rev. Clin. Oncol. 18 (5), 280–296. 10.1038/s41571-020-00462-0 33514910

[B8] Chua A. C. Klopcic B. Lawrance I. C. Olynyk J. K. Trinder D. (2010). Iron: an Emerging Factor in Colorectal Carcinogenesis. Wjg 16 (6), 663–672. 10.3748/wjg.v16.i6.663 20135713 PMC2817053

[B9] Dixon S. J. Lemberg K. M. Lamprecht M. R. Skouta R. Zaitsev E. M. Gleason C. E. (2012). Ferroptosis: an Iron-dependent Form of Nonapoptotic Cell Death. Cell 149 (5), 1060–1072. 10.1016/j.cell.2012.03.042 22632970 PMC3367386

[B10] Fanet H. Capuron L. Castanon N. Calon F. Vancassel S. (2021). Tetrahydrobioterin (BH4) Pathway: From Metabolism to Neuropsychiatry. Cn 19 (5), 591–609. 10.2174/1570159x18666200729103529 PMC8573752 32744952

[B11] Gai C. Yu M. Li Z. Wang Y. Ding D. Zheng J. (2020). Acetaminophen Sensitizing Erastin‐induced Ferroptosis via Modulation of Nrf2/heme Oxygenase‐1 Signaling Pathway in Non‐small‐cell Lung Cancer. J. Cel Physiol 235 (4), 3329–3339. 10.1002/jcp.29221 PMC13482668 31541463

[B12] Gao M. Monian P. Pan Q. Zhang W. Xiang J. Jiang X. (2016). Ferroptosis Is an Autophagic Cell Death Process. Cell Res 26 (9), 1021–1032. 10.1038/cr.2016.95 27514700 PMC5034113

[B13] Ghoochani A. Hsu E.-C. Aslan M. Rice M. A. Nguyen H. M. Brooks J. D. (2021). Ferroptosis Inducers Are a Novel Therapeutic Approach for Advanced Prostate Cancer. Cancer Res. 81 (6), 1583–1594. 10.1158/0008-5472.can-20-3477 33483372 PMC7969452

[B14] Grochot-Przeczek A. Dulak J. Jozkowicz A. (2012). Haem Oxygenase-1: Non-canonical Roles in Physiology and Pathology. Clin. Sci. (Lond). 122 (3), 93–103. 10.1042/cs20110147 21992109

[B15] Gryzik M. Asperti M. Denardo A. Arosio P. Poli M. (2021). NCOA4-mediated Ferritinophagy Promotes Ferroptosis Induced by Erastin, but Not by RSL3 in HeLa Cells. Biochim. Biophys. Acta (Bba) - Mol. Cel Res. 1868 (2), 118913. 10.1016/j.bbamcr.2020.118913 33245979

[B16] Harada T. Kagamiyama H. Hatakeyama K. (1993). Feedback Regulation Mechanisms for the Control of GTP Cyclohydrolase I Activity. Science 260 (5113), 1507–1510. 10.1126/science.8502995 8502995

[B17] Harrison P. M. Arosio P. (1996). The Ferritins: Molecular Properties, Iron Storage Function and Cellular Regulation. Biochim. Biophys. Acta (Bba) - Bioenerg. 1275 (3), 161–203. 10.1016/0005-2728(96)00022-9 8695634

[B18] Hassannia B. Vandenabeele P. Vanden Berghe T. (2019). Targeting Ferroptosis to Iron Out Cancer. Cancer Cell 35 (6), 830–849. 10.1016/j.ccell.2019.04.002 31105042

[B19] Hou W. Xie Y. Song X. Sun X. Lotze M. T. Zeh H. J. (2016). Autophagy Promotes Ferroptosis by Degradation of Ferritin. Autophagy 12 (8), 1425–1428. 10.1080/15548627.2016.1187366 27245739 PMC4968231

[B20] Jeught K. V. d. Xu H.-C. Li Y.-J. Lu X.-B. Ji G. (2018). Drug Resistance and New Therapies in Colorectal Cancer. Wjg 24 (34), 3834–3848. 10.3748/wjg.v24.i34.3834 30228778 PMC6141340

[B21] Jia L. Zhang P. Ci Z. Zhang W. Liu Y. Jiang H. (2021). Immune-Inflammatory Responses of an Acellular Cartilage Matrix Biomimetic Scaffold in a Xenotransplantation Goat Model for Cartilage Tissue Engineering. Front. Bioeng. Biotechnol. 9, 667161. 10.3389/fbioe.2021.667161 34150731 PMC8208476

[B22] Kang Y. Tiziani S. Park G. Kaul M. Paternostro G. (2014). Cellular protection Using Flt3 and PI3Kα Inhibitors Demonstrates Multiple Mechanisms of Oxidative Glutamate Toxicity. Nat. Commun. 5, 3672. 10.1038/ncomms4672 24739485 PMC4128233

[B23] Kim H.-L. Choi Y. K. Kim D. H. Park S. O. Han J. Park Y. S. (2007). Tetrahydropteridine Deficiency Impairs Mitochondrial Function inDictyostelium discoideumAx2. FEBS Lett. 581 (28), 5430–5434. 10.1016/j.febslet.2007.10.044 17976377

[B24] Kolinsky M. A. Gross S. S. (2004). The Mechanism of Potent GTP Cyclohydrolase I Inhibition by 2,4-Diamino-6-Hydroxypyrimidine. J. Biol. Chem. 279 (39), 40677–40682. 10.1074/jbc.m405370200 15292175

[B25] Kraft V. A. N. Bezjian C. T. Pfeiffer S. Ringelstetter L. Müller C. Zandkarimi F. (2020). GTP Cyclohydrolase 1/Tetrahydrobiopterin Counteract Ferroptosis through Lipid Remodeling. ACS Cent. Sci. 6 (1), 41–53. 10.1021/acscentsci.9b01063 31989025 PMC6978838

[B26] Li D. Li Y. (2020). The Interaction between Ferroptosis and Lipid Metabolism in Cancer. Sig Transduct Target. Ther. 5 (1), 108. 10.1038/s41392-020-00216-5 PMC7327075 32606298

[B27] Li Y. Yan H. Xu X. Liu H. Wu C. Zhao L. (2020). Erastin/sorafenib Induces Cisplatin-Resistant Non-small Cell Lung Cancer Cell Ferroptosis through Inhibition of the Nrf2/xCT Pathway. Oncol. Lett. 19 (1), 323–333. 10.3892/ol.2019.11066 31897145 PMC6923844

[B28] Liu J. Kuang F. Kroemer G. Klionsky D. J. Kang R. Tang D. (2020). Autophagy-Dependent Ferroptosis: Machinery and Regulation. Cel Chem. Biol. 27 (4), 420–435. 10.1016/j.chembiol.2020.02.005 PMC7166192 32160513

[B29] Liu J. Yang M. Kang R. Klionsky D. J. Tang D. (2019). Autophagic Degradation of the Circadian Clock Regulator Promotes Ferroptosis. Autophagy 15 (11), 2033–2035. 10.1080/15548627.2019.1659623 31441366 PMC6844535

[B30] Lu J. Zhao Y. Liu M. Lu J. Guan S. (2021). Toward Improved Human Health: Nrf2 Plays a Critical Role in Regulating Ferroptosis. Food Funct. 12 (20), 9583–9606. 10.1039/d1fo01036k 34542140

[B31] Mancias J. D. Wang X. Gygi S. P. Harper J. W. Kimmelman A. C. (2014). Quantitative Proteomics Identifies NCOA4 as the Cargo Receptor Mediating Ferritinophagy. Nature 509 (7498), 105–109. 10.1038/nature13148 24695223 PMC4180099

[B32] Mao C. Liu X. Zhang Y. Lei G. Yan Y. Lee H. (2021). DHODH-mediated Ferroptosis Defence Is a Targetable Vulnerability in Cancer. Nature 593 (7860), 586–590. 10.1038/s41586-021-03539-7 33981038 PMC8895686

[B33] Parseghian C. M. Napolitano S. Loree J. M. Kopetz S. (2019). Mechanisms of Innate and Acquired Resistance to Anti-EGFR Therapy: A Review of Current Knowledge with a Focus on Rechallenge Therapies. Clin. Cancer Res. 25 (23), 6899–6908. 10.1158/1078-0432.ccr-19-0823 31263029 PMC6891150

[B34] Pickert G. Lim H.-Y. Weigert A. Häussler A. Myrczek T. Waldner M. (2013). Inhibition of GTP Cyclohydrolase Attenuates Tumor Growth by Reducing Angiogenesis and M2-like Polarization of Tumor Associated Macrophages. Int. J. Cancer 132 (3), 591–604. 10.1002/ijc.27706 22753274

[B35] Radulescu S. Brookes M. J. Salgueiro P. Ridgway R. A. McGhee E. Anderson K. (2012). Luminal Iron Levels Govern Intestinal Tumorigenesis after Apc Loss *In Vivo* . Cel Rep. 2 (2), 270–282. 10.1016/j.celrep.2012.07.003 22884366

[B36] Soula M. Weber R. A. Zilka O. Alwaseem H. La K. Yen F. (2020). Metabolic Determinants of Cancer Cell Sensitivity to Canonical Ferroptosis Inducers. Nat. Chem. Biol. 16 (12), 1351–1360. 10.1038/s41589-020-0613-y 32778843 PMC8299533

[B37] Stockwell B. R. Friedmann Angeli J. P. Bayir H. Bush A. I. Conrad M. Dixon S. J. (2017). Ferroptosis: A Regulated Cell Death Nexus Linking Metabolism, Redox Biology, and Disease. Cell 171 (2), 273–285. 10.1016/j.cell.2017.09.021 28985560 PMC5685180

[B38] Stoyanovsky D. A. Tyurina Y. Y. Shrivastava I. Bahar I. Tyurin V. A. Protchenko O. (2019). Iron Catalysis of Lipid Peroxidation in Ferroptosis: Regulated Enzymatic or Random Free Radical Reaction? Free Radic. Biol. Med. 133, 153–161. 10.1016/j.freeradbiomed.2018.09.008 30217775 PMC6555767

[B39] Tang D. Chen X. Kang R. Kroemer G. (2021). Ferroptosis: Molecular Mechanisms and Health Implications. Cel Res 31 (2), 107–125. 10.1038/s41422-020-00441-1 PMC8026611 33268902

[B40] Thöny B. Auerbach G. Blau N. (2000). Tetrahydrobiopterin Biosynthesis, Regeneration and Functions. Biochem. J. 347 (Pt 1), 1–16. 10.1042/bj3470001 10727395 PMC1220924

[B41] Torti S. V. Manz D. H. Paul B. T. Blanchette-Farra N. Torti F. M. (2018). Iron and Cancer. Annu. Rev. Nutr. 38, 97–125. 10.1146/annurev-nutr-082117-051732 30130469 PMC8118195

[B42] Torti S. V. Torti F. M. (2013). Iron and Cancer: More Ore to Be Mined. Nat. Rev. Cancer 13 (5), 342–355. 10.1038/nrc3495 23594855 PMC4036554

[B43] Trachootham D. Alexandre J. Huang P. (2009). Targeting Cancer Cells by ROS-Mediated Mechanisms: a Radical Therapeutic Approach? Nat. Rev. Drug Discov. 8 (7), 579–591. 10.1038/nrd2803 19478820

[B44] Varghese F. Bukhari A. B. Malhotra R. De A. (2014). IHC Profiler: an Open Source Plugin for the Quantitative Evaluation and Automated Scoring of Immunohistochemistry Images of Human Tissue Samples. PLoS One 9 (5), e96801. 10.1371/journal.pone.0096801 24802416 PMC4011881

[B45] Wang H. (2020). MicroRNAs and Apoptosis in Colorectal Cancer. Int. J. Mol. Sci. 21 (15), E5353. 10.3390/ijms21155353 32731413 PMC7432330

[B46] Wang R. Su Q. Yin H. Wu D. Lv C. Yan Z. (2021). Inhibition of SRSF9 Enhances the Sensitivity of Colorectal Cancer to Erastin-Induced Ferroptosis by Reducing Glutathione Peroxidase 4 Expression. Int. J. Biochem. Cel Biol. 134, 105948. 10.1016/j.biocel.2021.105948 33609745

[B47] Wang R. Xing R. Su Q. Yin H. Wu D. Lv C. (2021). Knockdown of SFRS9 Inhibits Progression of Colorectal Cancer through Triggering Ferroptosis Mediated by GPX4 Reduction. Front. Oncol. 11, 683589. 10.3389/fonc.2021.683589 34336668 PMC8322952

[B48] Xia Y. Liu S. Li C. Ai Z. Shen W. Ren W. (2020). Discovery of a Novel Ferroptosis Inducer-Talaroconvolutin A-Killing Colorectal Cancer Cells *In Vitro* and *In Vivo* . Cell Death Dis 11 (11), 988. 10.1038/s41419-020-03194-2 33203867 PMC7673992

[B49] Xie Y.-H. Chen Y.-X. Fang J.-Y. (2020). Comprehensive Review of Targeted Therapy for Colorectal Cancer. Sig Transduct Target. Ther. 5 (1), 22. 10.1038/s41392-020-0116-z PMC7082344 32296018

[B50] Xie Y. Hou W. Song X. Yu Y. Huang J. Sun X. (2016). Ferroptosis: Process and Function. Cell Death Differ 23 (3), 369–379. 10.1038/cdd.2015.158 26794443 PMC5072448

[B51] Xu F. Ye M. L. Zhang Y. P. Li W. J. Li M. T. Wang H. Z. (2020). MicroRNA‐375‐3p Enhances Chemosensitivity to 5‐fluorouracil by Targeting Thymidylate Synthase in Colorectal Cancer. Cancer Sci. 111 (5), 1528–1541. 10.1111/cas.14356 32073706 PMC7226198

[B52] Xue J. Yu C. Sheng W. Zhu W. Luo J. Zhang Q. (2017). The Nrf2/GCH1/BH4 Axis Ameliorates Radiation-Induced Skin Injury by Modulating the ROS Cascade. J. Invest. Dermatol. 137 (10), 2059–2068. 10.1016/j.jid.2017.05.019 28596000

[B53] Yang W. S. SriRamaratnam R. Welsch M. E. Shimada K. Skouta R. Viswanathan V. S. (2014). Regulation of Ferroptotic Cancer Cell Death by GPX4. Cell 156 (1–2), 317–331. 10.1016/j.cell.2013.12.010 24439385 PMC4076414

[B54] Yang W. S. Stockwell B. R. (2016). Ferroptosis: Death by Lipid Peroxidation. Trends Cel Biol. 26 (3), 165–176. 10.1016/j.tcb.2015.10.014 PMC4764384 26653790

[B55] Yang W. S. Stockwell B. R. (2008). Synthetic Lethal Screening Identifies Compounds Activating Iron-dependent, Nonapoptotic Cell Death in Oncogenic-RAS-Harboring Cancer Cells. Chem. Biol. 15 (3), 234–245. 10.1016/j.chembiol.2008.02.010 18355723 PMC2683762

[B56] Ye S. Xu M. Zhu T. Chen J. Shi S. Jiang H. (2021). Cytoglobin Promotes Sensitivity to Ferroptosis by Regulating p53‐YAP1 axis in colon Cancer Cells. J. Cel Mol Med 25 (7), 3300–3311. 10.1111/jcmm.16400 PMC8034452 33611811

[B57] Ye Z. Hu Q. Zhuo Q. Zhu Y. Fan G. Liu M. (2020). Abrogation of ARF6 Promotes RSL3-Induced Ferroptosis and Mitigates Gemcitabine Resistance in Pancreatic Cancer Cells. Am. J. Cancer Res. 10 (4), 1182–1193. 32368394 PMC7191101

[B58] Zhang Y. Kong Y. Ma Y. Ni S. Wikerholmen T. Xi K. (2021). Loss of COPZ1 Induces NCOA4 Mediated Autophagy and Ferroptosis in Glioblastoma Cell Lines. Oncogene 40 (8), 1425–1439. 10.1038/s41388-020-01622-3 33420375 PMC7906905

[B59] Zhang Z. Xie X. Yao Q. Liu J. Tian Y. Yang C. (2019). PPARδ Agonist Prevents Endothelial Dysfunction via Induction of Dihydrofolate Reductase Gene and Activation of Tetrahydrobiopterin Salvage Pathway. Br. J. Pharmacol. 176 (16), 2945–2961. 10.1111/bph.14745 31144304 PMC6637045

[B60] Zhao Y. Li Y. Zhang R. Wang F. Wang T. Jiao Y. (2020). The Role of Erastin in Ferroptosis and its Prospects in Cancer Therapy. Ott 13, 5429–5441. 10.2147/ott.s254995 PMC7295539 32606760

