# Supplementary material for: Blockade of GCH1/BH4 Axis Activates Ferritinophagy to Mitigate the Resistance of Colorectal Cancer to Erastin-Induced Ferroptosis
Source: Front Cell Dev Biol. 2022 Feb 10;10:810327. doi: 10.3389/fcell.2022.810327 (PMC8866854; doi:10.3389/fcell.2022.810327)
Supplement: Supplementary file 1 [file DataSheet1.docx]

Supplementary Material

# Supplementary Data

- Supplementary Figures S1-4
- Supplementary Table S1-2

# Supplementary Figures and Tables

##
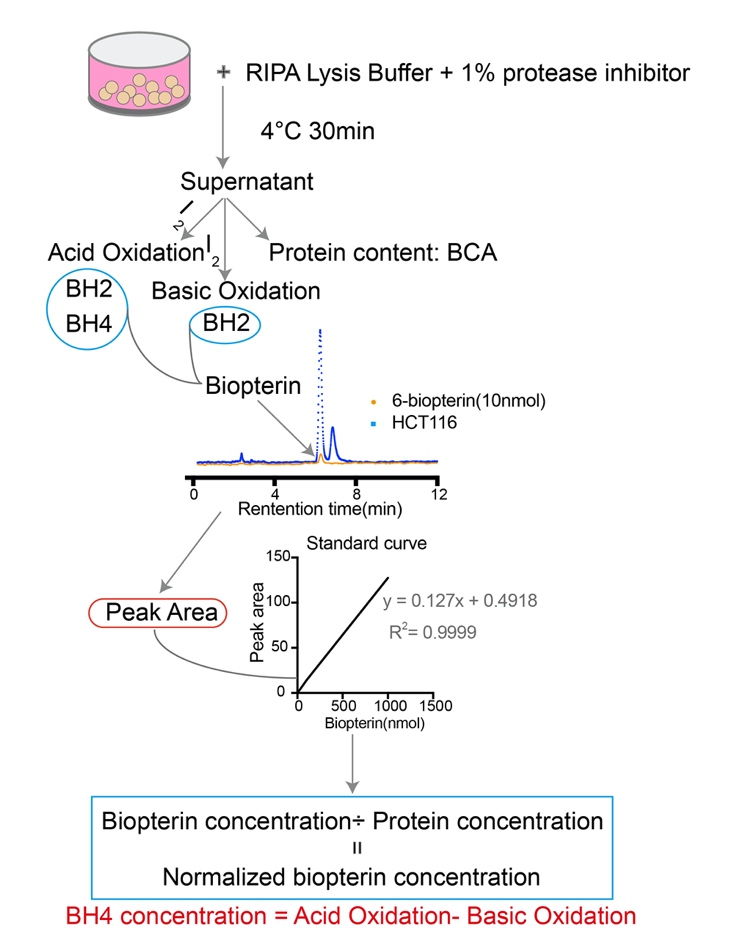
Supplementary Figures

**Supplementary Figure 1.** Flow chart of detection the content of cellular BH4 by HPLC. The flow path of detecting oxidized species of BH4 or total pterin by HPLC. First, culture cell in 10 cm dish for 24-48 h and lyse cells on ice for 30 min by RIPA (supplemented 1% protease inhibitor). Then collect the supernatant after centrifuged at 13,300 rpm at 4℃ for 30min. Besides, prepare acid oxidation and basic oxidation fluid (1N HCL/1N NaOH+1% I2+2% KI) in advance. Separate the cellular lysate into three parts: 100µl for acid oxidation (add 50 µl acid oxidation buffer), 100 µl for basic oxidation (add 50 µl basic oxidation buffer) and 10 µl for protein level assayed by BCA kit. After 1 h oxidation reaction, add 10 µl HPLC water (to acid oxidation) or 10 µl 5-N HCL (to basic oxidation) and add 5 µl 10% ascorbic acid to neutralize oxides. Again, collect the supernatant as before. Inject 20 µl supernatant into HPLC system (mobile phase: methanol 5% / 50 mmol·L−1 potassium phosphate buffer 95%, pH = 3.0). Collect the measured signals by fluorescence detector (λ excitation = 350 nm and λ emission = 440 nm). Afterwards, compare to external correction standard of 6-biopterin, we calculate the content of acid/basic oxidative production and normalized it to protein levels. Finally, BH4 = Acid oxidative production concentration-basic oxidative concentration; total pterin = acid oxidative production concentration.

**
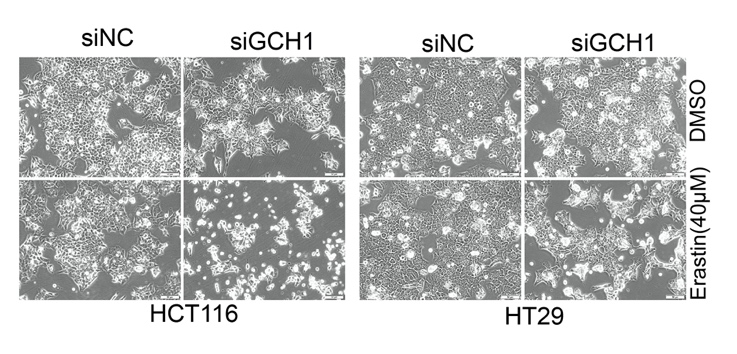
**

**Supplementary Figure 2.** Images of cell culture during erastin treatment in CRC cells. Microphotographs showing siNC or siGCH1 transfected CRC cells after exposure to DMSO or 40 µM erastin for 24 h. The bars in cell images indicate 50 µM.

**
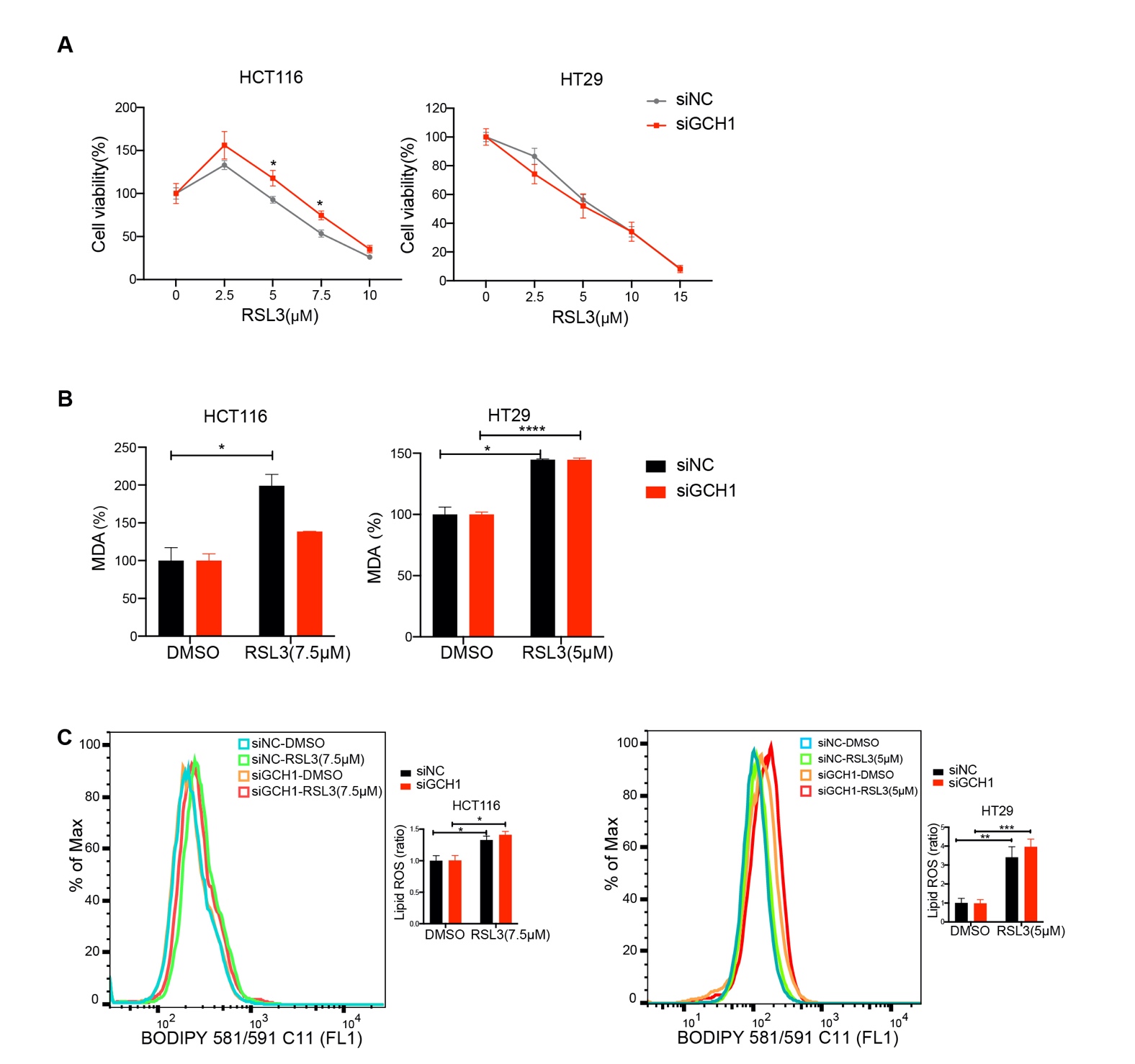
**

**Supplementary Figure 3.** Silencing GCH1/BH4 metabolism changes none of RSL3-induced ferroptosis segments in CRC cells. (**A**) Cell viability of the indicated cell treating with RSL3 for 24 h. **B**-**C**, lipid peroxidation detected by MDA assay kit (**B**) or BODIPY-C11 staining (**C**) in the indicated cells (The ratio of lipid ROS normalized to DMSO treating siNC cells). The error bars represent standard deviation from at least three replicates (#p < 0.05, compared between siNC and siGCH1 by unpaired t-test) (*p < 0.05, **p < 0.01, ***p < 0.001, ****p < 0.0001, compared between the two groups by unpaired t-test).

**
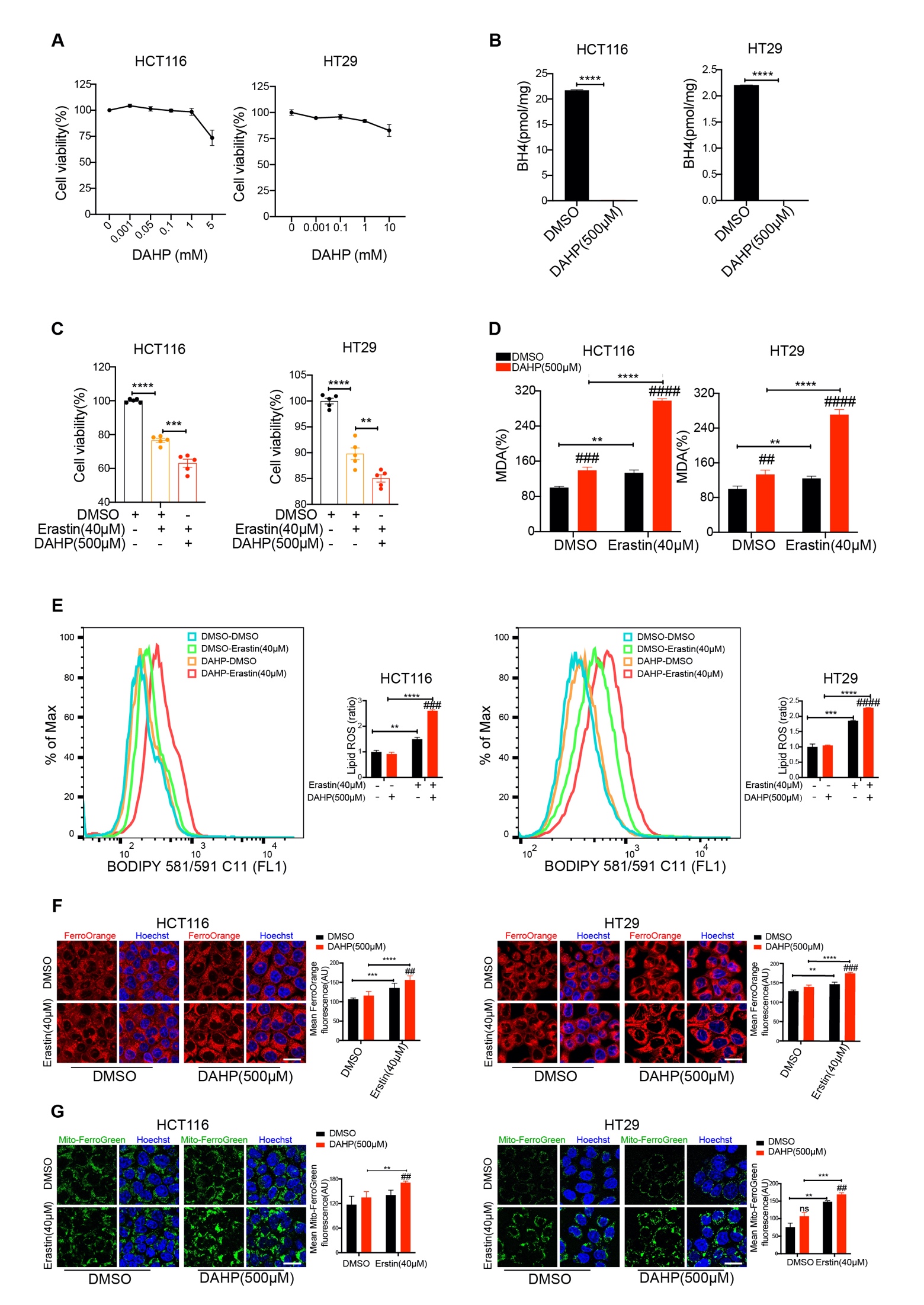
**

**Supplementary Figure 4.** Pharmacological inhibition of GCH1/BH4 metabolism and erastin induction synergistically induced ferroptosis in CRC cells. (**A**) The cell viability reflection to different doses of DAHP administration. (**B**) BH4 levels under 500µM DAHP treatment compared to DMSO in CRC cells. C. Synthetic lethality induced by 500µM DAHP and 40µM erastin treatment in HCT116 and HT29 cells. (**D**, **E**) Synergistic induction of Lipid peroxidation production by 500 µM DAHP and 40 µM erastin in HCT116 and HT29 cells. Lipid peroxidation was detected by MDA assay kit (**D**) or BODIPY-C11 staining (**E**) (The ratio of lipid ROS normalized to single DMSO treating cells). F-G, combination DAHP and erastin enhanced intracellular Fe^2+^(**F**) and mitochondrial Fe^2+^ (**G**) detected by FerroOrange and MitoferroGreen, separately. Scale bar, 20µM. The error bars represent standard deviation from at least three replicates (##p < 0.01, ###p < 0.001, ####p < 0.0001, ns, not significant, compared between siNC and siGCH1 by unpaired t-test) (**p < 0.01, ***p < 0.001, ****p < 0.0001, compared between the groups by unpaired t-test).

**Supplementary Table 1.** Primers pairs for ferroptotic genes expression analysis.

| **Gene** | **Forward primer** | **Reverse primer** |
| --- | --- | --- |
| GCH1 | GCTGTAGCAATCACGGAAGC | CACCTCGCATTACCATACACA |
| xCT | CCCAGATATGCATCGTCCTT | GCAACCATGAAGAGGCATGT |
| GPX4 | GGAGCCAGGGAGTAACGAAG | GACGGTGTCCAAACTTGGTG |
| HO1 | CCAGGCAGAGAATGCTGAGTTC | AAGACTGGGCTCTCCTTGTTGC |
| FTH1 | TGAAGCTG CAGAACCAACGAGG | GCACACTCCATTGCATTCAGCC |
| TFR1 | ATCGGTTGGTGCCACTGAATGG | ACAACAGTGGGCTGG CAGAAAC |
| DMT1 | AGCTCCACCATGACAGGAACCT | TGGCAATAGAGCGAGTCAGAACC |
| ferroportin | GAGACAAGTC CTGAATCTGTGCC | TTCTTGCAGCAACTGTGTCACAG |
| ALOX15 | AGCCTGATGGGAAACTCTTG | AGGTGGTGGGGATCCTGT |
| ACSL4 | TCTGCTTCTGCTGCCCAATT | CGCCTTCTTGCCAGTCTTTT |
| NCOA4 | ACAGTTGCATAAGCCGTCACC | TGAGCCTGCTGTTGAAGTGTC |
| LC3 | AGGCTGCGGGCTGAGGAGAT | TCTTTGTTCGAAGGTGCGGCG |
| ATG5 | AAAGATGTGCTTCGAGATGTGT | CACTTTGTCAGTTACCAACGTCA |
| ATG7 | ATGATCCCTGTAACTTAGCCCA | CACGGAAGCAAACAACTTCAAC |

**Supplementary Table 2.** SiRNA sequences used in this study.

| **siRNA** | **Sequences** |
| --- | --- |
| siGCH1#1 | GGAGCGCCTTACAAAACAA |
| siGCH1#2 | CTACCAGGAGACCATCTCA |
| siGCH1#3 | GTGTGAGCATCACTTGGTT |
